# Intra-body Networks and Molecular Communication Networks in Diagnostic Sciences

**DOI:** 10.7759/cureus.30399

**Published:** 2022-10-17

**Authors:** Prayas P Sarda, Sourya Acharya, Shreyash Huse, Yash Ghulaxe, Jay Chavada

**Affiliations:** 1 Department of Medicine, Jawaharlal Nehru Medical College, Datta Meghe Institute of Medical Sciences (Deemed to be University), Wardha, IND

**Keywords:** nanotechnology, diagnostic technology, molecular communication, biosensors, intra-body networks

## Abstract

Intra-body transmission networks are proposed to be composed of nanostructured or micro-sized detectors placed within the body to monitor health and distribute drugs. Transplanted biosensors are the potential options for monitoring the body for the detection of particular ailments and determining a diagnosis with the help of a doctor. Biological systems inside the body remain intricately integrated and interact primarily through biochemical interactions. Thus, the continuous communication performance and intra-body molecular nanonetworks coordinate essential functions within the human body. Spontaneous intra-body molecular nanonetworks, on the other hand, have yet to be investigated using sophisticated tools of information and communication concepts. We intend to understand the exquisite molecular networking that exists within us to design and develop pragmatic, effective interaction for evolving nanonetworks and also to lay the groundwork for the progression of groundbreaking diagnosis and therapeutic methodologies inspired by technological tools, which have the potential to be helpful in future nanotechnologies and bioinspired molecular communication (MC) applications.

## Introduction and background

The most promising communication paradigm for nanonetworks is molecular interactions, in which nanoparticles are used to record, transmit, and gather data. Another argument is that nanonetworks are created using proper tools on naturally existing occurrences, prospecting technical feasibility [1]. To develop molecular nanonetworks, examining specific molecular communication (MC) channels is necessary to provide the groundwork for molecular neuroscience. It is also essential to build nanobots and design information systems, both of which call for evolving efforts [2].

Luckily, the natural progression of the last several billions of years has provided us with such creative abilities and technologies. Gaining a greater understanding of the issues that can occur from communication failures opens the door for creating a new generation of information and communications technology (ICT)-inspired therapeutic techniques [3]. An essential advancement toward executing fully configured communication techniques for emerging connecting machine system implementation will also be identifying established intra-body molecular communication mechanisms and establishing the metadata philosophical underpinnings of these touchpoints [4]. Nanonetworks have a wide range of potential medicinal, ecological, and manufacturing uses. As a result of the fact that molecules, proteins, and DNA sequences naturally reside at the molecular scale, biomedical applications have the most potential in terms of utilizing these unique properties of nanonetworks. Furthermore, nanomachines might act as a bridge connecting biological mechanisms and large-scale electronic systems [5]. In intra-body networks, for example, a collection of nanosensors will gather information on the concentration of various substances or the presence of specific agents (such as cancer biomarkers) and remotely send it to the macroscopic scales. Intra-body networks are intended to make it possible to use cutting-edge, amazingly accurate patient-monitoring technologies [6].

Inter-individual physiological systems communicate with one another to carry out complex critical functions. Apart from their coexistence, any ecosystem comprises a mass of molecular and cellular structures that assemble to perform distinct biologically active compounds. They share the primary communication technique for carrying out certain activities, such as atomic data transfer via chemical sodium channels or simple action prospective propagation to alert target cells. The basic principles of molecular communication for living organisms can be accomplished inside one general framework of interpersonal and interatomic communication channels, although the driving mechanisms behind communications may change even in different structural components [7]. The networking community is still grappling with a significant difficulty regarding how bionic implants will interact. Several networking models have already been put forth to establish interaction at the nanoscale. Among them, molecular communication (MC), a biological phenomenon present in nature, is one of the most promising. Cells communicate using MC, which encrypts signals into molecules released by the transmitter and moved directly to the receivers [8].

The neural nanonetwork is a colossal network comprising nano-transceivers or synapses that span the entire body. The primary nerve network (PNN) and the central nervous network (CNN) are indeed the two key subnetworks. The CNN combines sensory data and sends motor functions to adhesion molecules, such as muscle cells and cell organelles. It is the body's primary processor. The sensory nerve subnetwork (SoNS) and the autonomic nervous subnetwork (ANS) are the two sections of the PNN. The SoNS gathers data from the sensors of the five senses and the brains. The ANS sends information to the CNN via sensory neurons. The CNN generates the motor outputs to muscles and glands via motor neurons. The PNN's primary role is to connect the CNN to the systems [9]. In medical and biological surveillance systems, using the human body as a transmission channel for electrical impulses opens up new possibilities for data transfer [10].

## Review

Networks for molecular communication: a general framework

The transmitter, channel, and reception are the primary operational blocks of a typical molecular nanonetwork with two nanobots or nano-nodes. These three functional blocks are in charge of the release, propagation, and receiving of activities. This broadcaster transmits a message, also known as little more than a carrier wave, that comprises the transmitted data to be transferred. By emitting particles into the interior in response to specific feedback and biochemical evidence-embedding framework, such as cellular accumulation and a compound category in quite an activity prospecting biochemical data transfer, and consequently the amplitude and frequency of electrochemical impulses in a central nervous system transmission medium, this identical macromolecular combustion system generates a digital output. The recipient extracts the transmitted data from the perceived signal and collects the data coming. The nucleophile-binding pathway is a common absorption mechanism in tissue samples [11].

Nanoscale neuro spike communication channel

The neuronal molecular network, made up of network nodes (masses of nerve cell bodies), can aggregate information from different body parts, aggregating the same and providing the body's necessary feedback. A dispersed connection runs throughout the body and reaches toward the appendages. Neurons are electrically excitable sensory receptors designed to store, process, and send information via electrical and chemical signaling mechanisms, which are thought of as nervous building networks called nano-transceivers. The electrical polarization of the membrane structure changes when they receive messages from other neurons or sensory cells. Electromotive force is distributed throughout the cytosol and merged at the axon's foundation, having caused nerve impulses to be generated. The neuroendocrine cell responds to increases in the particular compound in the extracellular fluid via a signaling pathway and provides homeostatic control of the accumulation of substances within the cell. These excitons are then transmitted through the axon and arrive at its divisions, where the neurotransmitter interacts with other neurons via synaptic or semiconducting ties between serotonergic neurons and associates with the project molecules. The results of a hormone's release, as well as the activity of the target cells, suppress its subsequent release. The hormonal control loop can occur at any level, during hormonal chemical modification, and when held substances are released [12].

Network of intra-body molecular nanonetworks

We must analyze the relationship involving neurological, circulatory, and hormonal mobile ad hoc networks based on intermolecular nanoscale networks to comprehend molecular interaction across diverse intra-body small-cell networks. The vasoconstrictor center, a particular area of the brainstem throughout the neurological process despite its advanced state, is crucial to the communication between the cardiac chemical nanonetwork and the central nervous system subnetwork. It gives excitation inputs to the heart via sympathetic nerve fibers if there is a need to increase heart rate and contractilities, such as during muscular exertion or even other types of stress. Instead, it sends signals to the neurological system, which sends parasympathetic impulses to the heart, slowing its beat and contractility and enabling the heart to circulate blood as needed [13].

**Figure 1:**
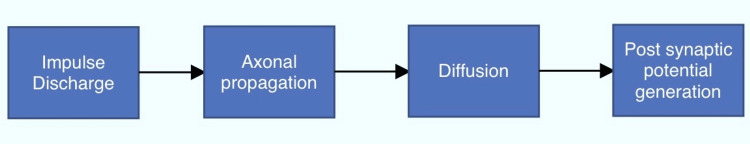
Neuro spike communication

An extremely large-scale neuron structure, the neurological neuronal network assembles and transmits information between various body parts. Synthetic and electrical flagging, also known as "neuron spike communication," is how neurons communicate with one another. As portrayed in Figure 1, an overall brain-flagging pathway comprises three basic designs: a presynaptic neuron, a synaptic cleft, and a postsynaptic neuron [14]. The data recorded in electrical stimuli travels via the axon of a presynaptic neuron, which transmits information through re-renting synapses, which are distinct types of substance molecules, into the synaptic split. These synapses are then received by the postsynaptic neuron as they spread over the synaptic divide. The real channel model of solitary, single-input single-output (SISO) neuron spike transmission is accepted, and attention is given on the influence of inter-symbol interference (ISI) on the SISO splitter's applicable data rate [15]. Honestly, there exist one primary neurotransmitter and many co-transmitters between two neurons. In this manner, the multiple-input single-output (MISO) correspondence model is displayed [16].

Although a whole neuron spike communication channel is considered, several analyses deconstruct the channel's discrete components. Within the axonal direct, for instance, a compromise between data rate and energy productive transmission is addressed [17]. A practical model for vesicle discharge, which includes the impacts of action potential (AP) width variety, is proposed [18]. Besides, a recipient plan in an apprehensive organization considering the changeability in the life structures of dendrites is given [19]. The ordered environment has also been used to analyze neuron spike correspondence. A neuronal time division multiple access (TDMA) enhancement issue is created to find the appropriate planning [20]. A queueing model for worried nanonetworks is also put forth, which acknowledges the postponement of neuronal organization. Instead of focusing solely on data capacity, the cautious nanonetwork is an ideal data-handling structure for energy productivity [21,22]. Intra-body nanosensor networks collect climate data, which is then processed, handled, and transmitted to associated organs by the sensory system. Various studies have been conducted to evaluate these encoding schemes, including ones that consider discrete parallel and recurrence coding, interspike span codes, rate decoders, and interspike stretched decoders [23,24]. Then again, in examinations, for example, improvement reaction bends are fitted to enter/yield relationships of neurons regarding hypothetical data boundaries. Be that as it may, more endeavors are expected to arrive at the productivity of brain-encoding and brain-translating instruments [25].

Cardiovascular Nanonetworks

**Figure 2:**
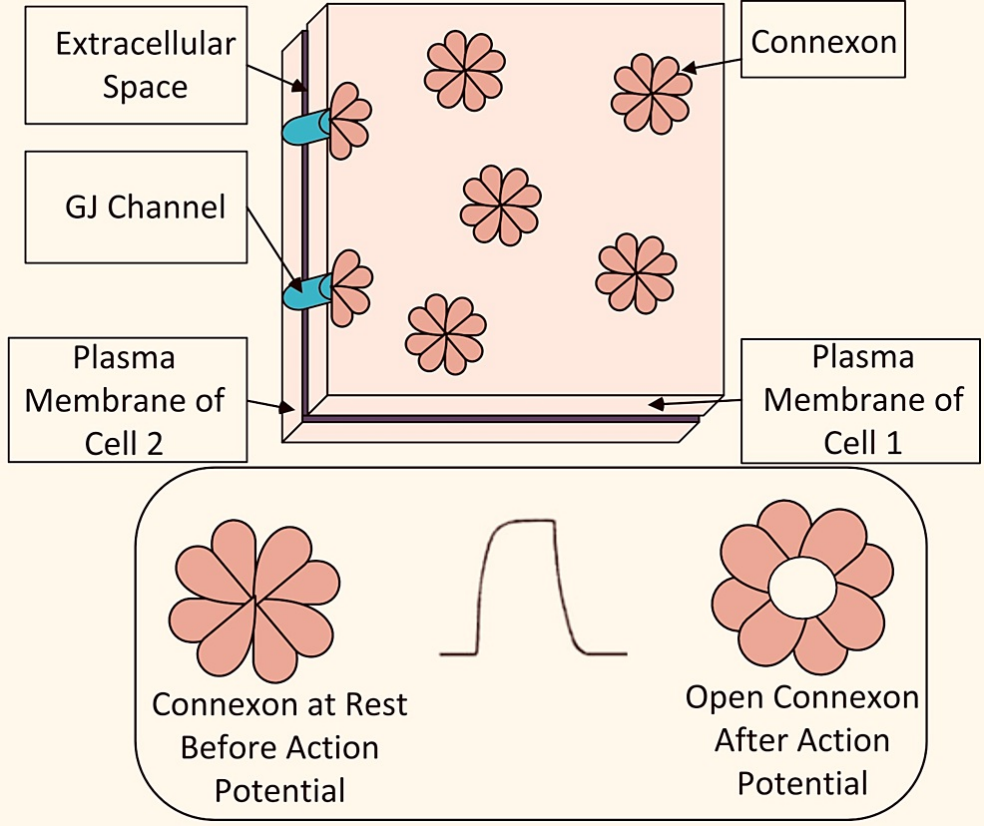
GJ communication Action potential causes the connexin hemichannels to open and start diffusion between the cells GJ: gap junction

Cardiomyocytes and cardiovascular pacemaker cells are two of the most important types of cells in the heart [26]. Cardiovascular pacemaker cells are found in limited region of the heart and are responsible for rapidly producing and multiplying APs to cardiomyocytes, which cause the heart to beat rapidly. A medium for AP movement is provided by cell-spanning structural permeability gap junctions (GJs) between the various types of cardiac cells [27]. Connexon-based channels in the GJ connect two cells as shown in Figure 2. Connexons remain closed in the resting state, but when an AP appears, they open and cause a particle to spread toward the collector cell, causing the AP to be transmitted to the next cell. There is a concentration of traditional GJ correspondence [28]. A closed structure articulation for channel limit and the association between the limit decrease and a few heart diseases are considered [29].

Endocrine Nanonetwork

The endocrine system consists of several organs that release chemicals into the bloodstream to regulate the functions of cells [30]. Groupings of various components found in tissues are what enable chemical transmission. The endocrine system then adds the due amount of hormones to the circulatory system to maintain the body's homeostasis [31]. Chemical production depends on blood distribution and Brownian motion inside the circulatory system. Because the goal cell can respond to a chemical, gathering the chemicals is unique. Displaying the entire endocrine system from an ICT perspective is an open research area with enormous implications. In the paradigm of the endocrine nanonetwork, hormones are thought of as modified atomic data transporters. The proposed structure is where various enhancements trigger the nervous system's center to signal the blood's arrival of hormones [32]. The reciprocal relationship between neuroendocrine and safe frameworks is being investigated [33]. For microorganisms to have correspondence, employing chemicals is shown [34,35].

Molecular communication and information science for life sciences

ICT-Based Understanding of Human Diseases

In this paper, we relate some anxiety disorders to the ICT-based anxious nanonetworks' bounds. Alzheimer's disease is caused by a breakdown and loss of synaptic connection, which impair memory encoding and retrieval [36]. Multiple sclerosis slows down data transmission because it causes the myelin to be destroyed along the neuron's axon [37]. Parkinson's disease is caused by the degeneration of dopamine-producing neurons, which control how vesicles, a type of data transporter, enter other types of neurons [38]. The degradation or death of the upper and lower engine neurons results in the specific engine neuron disorders amyotrophic lateral sclerosis, progressive bulbar sclerosis, and primary lateral sclerosis. These diseases result in correspondent link failure in the nervous nanonetwork, which impairs the brain's ability to regulate basic muscle movements such as walking, talking, swallowing, and breathing [39].

In cardiovascular framework illnesses, heart arrhythmia is one of the most widespread groups of heart disorders that give patients erratic heartbeat patterns. They uncovered this entire set of problems using regular electrocardiographic methods, which are physically done by a professional and hence prone to human error [40]. Consequently, demonstrating these illnesses may find ICT-based distinguishing proof and treatment of these circumstances.

In endocrine disorders, although there have been a few studies on the biochemical cycles of various substances, the largest amount of writing has focused on the movements of insulin inside the body, making it conceivably the main subject of an ICT-based study. In light of the queueing idea, recent research suggests a novel correspondence-based method for transporting insulin-injected Overabundance 4. Type 2 diabetes develops due to insulin resistance in tissue cells, which renders them unable to use sugar effectively and produces high glucose levels. Using insulin siphons can improve the existing diabetes finding, medications, and executives by understanding the MC disappointment that reduces the sugar take-up despite the presence of insulin receptors on tissue cells [41,42].

Future research avenues

Even in aberrant settings, molecular processes allow the mammalian body to work together. However, in some disorders, living organisms cannot overcome issues caused by external stimuli or by the body itself. In this research, we focus on unsolved or incompletely recognized impacts on human health connected to the faulty or insufficient functioning of cardiac, endocrine, neurological, and nanosensor small-cell networks [43]. Healthcare has primarily focused on mobilizing therapeutically essential organ processes in the body. The truth is that cardiac and brain diseases need to be treated immediately because they are crucial to survival and control many other critical organ functions in the body [44]. As a result, significant emphasis is placed on cardiovascular surgery, neurosurgery, hormones, and diabetic illnesses, among other things. Many ailments, including diabetes, heart failure, cardiac arrhythmias, renal failure, and different cancers, can now be treated thanks to significant medical developments. Numerous studies have been conducted on chemical communication, particularly from the angles of propagation media, diffusion, and the noise sources used in the distribution process. Several bioinspired solutions are explored if signal propagation necessitates a fixed physical link, i.e., wired or wireless communication. Light propagation, spore, dust, and pheromones are discussed. The other group looks into the capillary flow network and nerve cell interaction [45].

## Conclusions

This study presented an altogether clever impression of ideal atomic correspondence models to set out the underpinnings of the analysis of atomic data and correspondence. The body is an embodiment of nuclear flagging, which is being studied to develop and optimize nanonetworks. To pinpoint and separate the fundamentals of inter- and intrainformation and corresponding science, we took the opportunity to analyze recent research on intra-body nanonetworks and MC and highlight outstanding issues that need to be addressed. We developed a method to locate critical thresholds in light of this analysis by linking the ICT-based MC channel's attributes to its physical and chemical traits. Finally, we highlighted the ability of subatomic information and connection research in biological sciences, focusing on its applications in developing ICT-based processes for diagnosing and treating infections caused by flaws in intra-body nanonetworks. Nanobots, also known as straightforward nanomethods that can be used for a wide range of tasks, have been a crucial part of applications for nanoscale communication at the nanoscale or simple nanomethods that can be used for a wide range of tasks, particularly in applications for the Internet and nanoscience. In conclusion, research into the fundamental interconnections between people and artificial intelligence (AI), as well as the importance of these links in sustaining the integrity of all biological systems via nanonetwork connections, expands and enhances the ICT sector. As a result, it is projected that ICT will play a crucial role in developing nanomolecular communication systems that are modeled after the biological network of people and open the way for designing ICT-based treatments for interpersonal diseases.
